# Exploring the Competencies of Japanese Expert Nurse Practitioners: A Thematic Analysis

**DOI:** 10.3390/healthcare9121674

**Published:** 2021-12-03

**Authors:** Mari Igarashi, Ryuichi Ohta, Yasuo Kurita, Akinori Nakata, Tsutomu Yamazaki, Harumi Gomi

**Affiliations:** 1 Graduate School of Medicine, International University of Health and Welfare, Tokyo 107-8402, Japan; nakataa@iuhw.ac.jp (A.N.); yama@iuhw.ac.jp (T.Y.); hgomi@iuhw.ac.jp (H.G.); 2 Community Care, Unnan City Hospital, Unnan 699-1221, Japan; ryuichiohta0120@gmail.com; 3 School of Medicine, International University of Health and Welfare, Narita 286-8686, Japan; kurita-yasuo@iuhw.ac.jp

**Keywords:** nurse practitioner, Japanese context, competency, quality of life, clinical contexts

## Abstract

Nurse practitioners (NPs) provide medical care equivalent to that of physicians and facilitate access to healthcare. Although Japan’s first NP graduated in 2010, how Japanese expert NPs work effectively in clinical contexts is yet to be investigated. We aimed to identify the competencies that make expert NPs in Japan effective. Twelve Japanese expert NPs were purposely selected. The average age of the participants was 44.8 years, average NP experience was 7.5 years, and eight participants were women. Semi-structured interviews were conducted online from March to May 2021. Interviews were recorded, transcribed verbatim, and analyzed using thematic analysis. Thematic analysis revealed five themes: working in physicians’ contexts, interprofessional collaboration, involvement in nurses’ work, contribution to healthcare, and personal qualities for effective working. Japanese expert NPs can function effectively in clinical settings by flexibly and humbly collaborating with other medical professionals who have autonomous positions. They can improve the quality of healthcare by proposing practical solutions to problems faced by patients and medical organizations. These explored competencies can be applied to other aging and more complex societal contexts, and in updating the required competencies of Japanese NPs.

## 1. Introduction

Among the various issues faced by the world today, challenges related to healthcare delivery systems are on the rise [1]. The increased healthcare challenges are due to several factors, including the increasing number of patients with chronic and complex diseases as the population ages, the advancement of medical care, and the diversification of medical needs [2]. The challenge of securing and allocating sufficient healthcare resources has led many healthcare systems to review the roles of healthcare professionals in an attempt to identify methods of building new collaboration systems that utilize resources more effectively [3,4]. There is a growing interest among healthcare professionals regarding substituting physicians with nurses to overcome the challenges of limited healthcare resources [5]. More specifically, advanced practitioner nurses, who receive additional education in medical skills in graduate school, have been considered effective candidates for substituting physicians [6]. There are four types of advanced practitioner nurses: nurse midwives, nurse anesthetists, clinical nurse specialists, and nurse practitioners (NPs) [7]. Among them, the role of NPs is defined as integrating nursing and physician skills to diagnose and manage patients in primary and acute healthcare settings and provide continuity of care for patients with chronic illnesses [6]. In the United States (US), NPs can provide non-surgical medical services, such as performing diagnoses and prescribing medications [7]. Currently, there are approximately 325,000 NPs in the US, 90% of whom provide primary care [8]. Canada, the United Kingdom, Australia, New Zealand, the Netherlands, and Singapore have also introduced NP programs [3,4]. Previous studies have shown that NPs are at least as competent as physicians in improving access to care, reducing waiting times, preventing serious illness, and improving patient satisfaction; furthermore, they have no negative impact on patient outcomes [9]. Following the world trends in NP education, Japan began training NPs in 2008 [10]. The increase in medical needs due to a rapidly aging society and the uneven distribution of physicians in rural and urban regions in Japan have driven the need for NPs to assist in improving the healthcare delivery system. Despite the absence of a legal framework regulating the medical practice of NPs, educational institutions have been training motivated Japanese nurses [11,12,13]. By the end of March 2021, approximately 10 years after the first NP graduated in Japan in 2010, a total of 582 NPs had received certification from the Japanese Organization Nurse Practitioner Faculties (JONPF) [10]. However, without a common understanding of the role of NPs among healthcare professionals, it is difficult to standardize and assure the quality of NP educational programs. To improve the present situation, a clear understanding of the work and effectiveness of NPs, based on the actual performance of expert NPs in Japan, is required.

Although the overall clinical performance of NPs needs clarification, their work has been documented as clinical reports on individuals’ work activities, case reports, narrative reviews, and reports of studies conducted in single institutions [14,15]. Ito et al. [16] conducted a qualitative study, using Bloom’s taxonomy as a framework, for the seven abilities proposed by JONPF as necessary for NPs. The research suggested that NPs utilize their knowledge, skills, and attitudes in interprofessional collaboration. However, the results of the study may not be generalizable because it was conducted at a single institution and the participants had little NP experience. Therefore, our research aimed to investigate the question, “What competencies are needed for experienced NPs to work effectively in clinical practice in Japan?” To answer this question, we investigated the activities of NPs from a nationwide perspective, as these activities represent the competencies of Japanese NPs who effectively perform clinical duties. Clarifying NP activities will promote understanding of an NP’s role among healthcare professionals, provide data for examining NP competencies in the Japanese context, and contribute to the standardization of pre- and post-graduate education for NPs.

Furthermore, the effective use of NPs in Japan, which is a country with the oldest population in the world, can inform the reconstruction of the healthcare delivery system in aging societies. The roles and activities of NPs may be affected not only by their competencies, but also by the cultures and traditions in healthcare. The competencies of NP experts have been defined in research literature as their roles and working styles for the improvement of healthcare [17].

## 2. Materials and Methods

A purposive sample of NPs certified by the JONPF was recruited with the aim of identifying their competencies to perform clinical tasks effectively. From February to March 2021, we used PubMed™, Google Scholar™, and CINAHL™; the Central Journal of Medicine, which contains the largest number of research articles in Japanese; and the Journal of the Japanese NPs, which is published by the academic association of NPs in Japan. The literature was searched using the keyword “the Japanese NPs”; 3 researchers checked the content of the literature, and 20 people with active duties as NPs were selected. Research requests were made in order of NPs with the most published activities, and interviews were conducted with those NPs who voluntarily provided written consent. Twelve interviews reached data saturation, and the study was terminated at that point.

### 2.1. Setting 

In 2008, when Japan began to consider expanding the certified fields of non-physician occupations, the Oita University of Nursing and Health Sciences started training NPs in its master’s program. Before entering the 2-year master’s program, NP students are required to have completed five years of clinical experience as a nurse. At the end of graduate school, students take the NP certification exam and become NPs. The NP certification is administered by the JONPF affiliated with the NP training graduate school [5]. The graduates proceed to find employment in a wide range of settings across Japan, including hospitals, home care, nursing homes, and remote islands.

### 2.2. Semi-Structured Interviews

Semi-structured interviews were conducted with the 12 NPs. The interviews were conducted using the online meeting system Zoom™ to reduce the risk of COVID-19 infection during the pandemic. Our interviews followed an interview guide distributed to the participants in advance. The interview guide consisted of four questions: (1) What kind of work do you do now as an NP? (2) What skills do you think are necessary to perform this job? Explain why these are necessary. (3) Please tell us an episode that explains why you need those abilities. (4) How do you think one can acquire these abilities? No particular problems were encountered regarding online interactions. NPs’ perceptions and opinions on matters related to these questions were recorded and translated verbatim.

### 2.3. Analysis 

No theory was used, and inductive thematic analysis was utilized to identify the comprehensive competencies of expert NPs in Japan [18]. The first author is a Japanese NP with clinical experience in critical care. The second author is a Japanese primary care physician who has worked with NPs in primary care settings and has experience in conducting qualitative analysis. The thematic analysis comprised six steps: familiarization with data, generating initial codes, identifying themes, revising themes, defining and naming themes, and writing the report [18]. The analysis was performed by the two researchers, using the triangulation method to ensure credibility. At the first step, the first and second authors carefully read the interview transcripts. At the second step, they independently coded the content based on repeated reading. At the third and fourth steps, to ensure dependability, the two authors consistently discussed and compared research materials and coding, and introduced, merged, deleted, and refined concepts and themes until mutual agreement was reached and no new concepts or themes were added, leading to theoretical saturation. At the fifth step, the themes and concepts were discussed and agreed upon by all authors. At the last step, only the final results were translated from Japanese to English for submission to an international journal.

### 2.4. Ethical Considerations 

Study participants were informed in advance that all data collected would be used for research purposes. Before participants provided written consent, they were informed of the purpose of the study, how the data would be used, and how their personal information would be protected. This study was approved by the Ethics Committee of the International University of Health and Welfare (Approval number: 20-Im-017).

## 3. Results

The mean age of the participants was 44.8 years (SD = 8.6); the mean years of NP experience was 7.5 (SD = 2.1); the mean years of clinical nursing experience, including NP experience, was 22 (SD = 7.0). Of the 12 participants, 8 were female. NPs in Japan work in two main practice domains: primary care and critical care; three NPs worked in primary care and the remaining nine worked in critical care. Interviews were conducted from 28 March to 11 May 2021 and took an average of 60 min per interviewee to complete. After the 12th interview, data saturation was reached, and data collection was terminated. All interviews were performed effectively, without Internet problems. Data were analyzed using thematic analysis and were categorized into five themes and 15 concepts (Table 1).

### 3.1. Working in Physicians’ Contexts 

#### 3.1.1. Understanding the Physician Context

NP duties, focused on patient care, required a deep understanding of the physician’s context. The participants emphasized the importance of understanding the ways physicians think and reason in order for clinical practice to facilitate medical treatment.

“I think it is important to be able to treat patients in emergency with common diseases and to have a strategy to deal with emergency cases. It is important to see patients with a clear understanding of clinical duties and to intervene from the same point of view as the physicians’.”

(NP9)

“First of all, it is the connection with the doctors. This is a critical aspect of teamwork in medical care. Every morning, we go on a round with the doctors. Through these rounds, we confirm the treatment plan for each patient, discuss necessary procedures and clinical decisions, and decide the daily schedule for patients.”

(NP4)

Understanding the physician’s context involves an understanding of evidence-based medicine and the practice of clinical reasoning. It is not only necessary from the medical perspective but also helps build a trusting relationship with physicians. 

#### 3.1.2. Comprehensive Support for Medical Practice 

NPs were expected to be as competent as physicians in providing medical care. They performed medical procedures, supported surgical operations, and worked to further improve their skills under the guidance of physicians.

“For example, I can administer antibacterial agents quickly, and can send the patient to the hospital by ambulance while reporting back to the doctor. I think NPs can respond quickly.”

(NP5)

“In the acute phase, there is not much focus on chronic diseases and poor control of underlying diseases. I hope to be able to help with disease prevention as an NP.”

(NP11)

NPs not only provide the same level of medical care as physicians but also play an important role in the transitional care of patients. In departments with a strong specialization component, NPs are expected to be more skilled than inexperienced physicians, and, thus, are considered more advanced medical service providers. Rather than simply overcoming the shortage of physicians and maintaining expected levels of service, NPs provide extensive support to patients and enhance medical safety. 

#### 3.1.3. Active Involvement in Physician Education 

To become a doctor in Japan, medical graduates have to undergo training as residents, during which time their knowledge is put into practice. Residents are usually taught by skilled physicians, but experienced NPs are seeing their role expanded by being entrusted with the education of younger physicians.

“I think most NPs are now teaching medical skills that involve the venous puncture procedure, such as peripherally inserting a peripherally inserted central catheter, securing arterial lines, and conducting arterial blood collection. NPs are already teaching residents. At our clinic, although there is a senior doctor who oversees all operations, I mainly intervene and tell the residents how they should do things, how to prepare necessary items, and how to read/interpret the ultrasound images.”

(NP1)

“I do not know if it is at the same level or of the same quality, but I think I can provide more information to nurses and other co-medics.”

(NP6)

Experienced NPs instruct residents about technology use in medicine, how to work in a medical setting, and how to collaborate with multiple professionals. The medical skills of NPs and their ability to teach and develop relationships with other professionals have led to their active involvement in physician education.

#### 3.1.4. Knowing the Limits of One’s Capabilities 

NPs believed that it was important to understand the risks involved in providing medical care, while simultaneously being aware of the limitations of their abilities and actively communicating these limitations to the doctors and other medical staff around them.

“I think we are excellent at performing procedures that otherwise are performed by physicians. Therefore, we need to rethink whether a procedure needs to be performed by physicians and if NPs with training and knowledge can intervene in such areas. However, there are many areas where I absolutely cannot intervene, and I leave those completely to the doctor.”

(NP1)

“It is crucial for me to clearly say what I can and cannot do and to be able to ensure patient safety.”

(NP9)

NPs recognize that any procedure without proper education or experience should not be performed. This emanates from a deep understanding of medicine and a strong awareness of patient safety. NPs also make efforts to dispel anxiety and concern about their activities by proactively communicating with those around them. In addition, they are aware of their limitations as medical practitioners, which provides clarity in their roles.

### 3.2. Interprofessional Collaboration 

#### 3.2.1. Bridging Multiple Professions 

NPs were aware of the gap that existed in the perceptions of multidisciplinary teams regarding the patients’ conditions and the management of their conditions. They felt that they needed to adjust to bridge this gap, and to this end, they frequently communicated with other professionals to facilitate patient care. NPs considered themselves to be a link in the medical care team, coordinating with various professionals to support their respective professions.

“The main professionals involved are nurses; ward nurses; infection control nurses; wound, ostomy, and continence nurses; clinical nurse specialists; physical therapists; occupational therapists; and most often social workers. During the daytime, we are very busy collaborating with these professionals and playing a key role in team medicine.”

(NP4)

“In surgery, I usually work as an assistant, but I also have to take care of the entire operation. If the nurse who is handling the instruments is young, I have to support her, and because the use of instruments changes depending on who the surgeon is, it is important to control such things to ensure smooth operation.”

(NP3)

“There is a considerable gap between what the doctor thinks and what the nurses on the frontline are trying to do for the patients and their families in the elderly care facilities. I think our role is to fill this gap.”

(NP5)

To mitigate any misunderstandings and communication gaps, NPs proactively share information with team members, assess the overall picture of the team, compensate for members’ weak points, and work to ensure that everything proceeds smoothly. NPs also ensure ethical decision-making for the patient and are aware of the mental state and working conditions of each medical professional.

#### 3.2.2. Taking Advantage of Diverse Contexts 

For NPs to work smoothly in the workplace, they needed to understand the contexts of diverse professions. While interacting and collaborating with various professionals, NPs were able to deepen their understanding of not only the doctors’ context but also that of other professions and ensured smooth collaboration with other professionals.

“I think it is the ability to collaborate because the idea is to know other professions, what other professionals are capable of, and to think about the kind of services I can provide because of this collaboration.”

(NP4)

“I think it is good for a person to know where she/he needs to be to maximize her/his growth in the team and to work hard in that position.”

(NP2)

NPs recognize the importance of interacting with multiple professionals and understanding different contexts. The participants felt that their roles as NPs would become clearer if they could promote multidisciplinary collaboration based on a deep understanding of the team environment.

#### 3.2.3. Humble Collaboration 

While working as NPs, the participants became more conscious of the quality of humility. They experienced the negative effects of assertiveness in multidisciplinary collaboration and understood the importance of suppressing the arrogance that comes with experience. Instead, they understood the need for displaying humility and empathy in conducting patient care and collaboration with other professionals.

“When you become an experienced nurse, you need to be humble and not look down on less experienced nurses. I think humility is important. If you are not humble, you will lose working opportunities.”

(NP7)

“I think that people will approach a person who is kind to others and not meddlesome. I think it is all about the attitude and tone of voice. If NPs do not consciously pay attention to this, they tend to be seen as strong. I think the way we talk changes depending on whether we know that we tend to be seen as strong.”

(NP1)

NPs believe that by being humble, they could collaborate with multiple professionals. As an NP, it is important to work humbly with other professionals to play a key role in facilitating multi-professional collaboration. One participant stated:

“If I had to say what is necessary, it would be a certain amount of cooperation. I do not think it is necessary to be extremely collaborative. It is okay if you are egotistical, but if you are not forthcoming and have a hard time interacting with others, I think it is difficult to be an NP in many ways.”

(NP11)

### 3.3. Involvement in Nurses’ Working 

#### 3.3.1. Communicating Medical Contexts 

Communication between doctors and nurses is directly related to the quality of medical care. However, in practice, there is a gap between doctors and nurses in their consideration and understanding of patients. This is due to differences in the expertise of doctors and nurses regarding diseases. Physicians tend to consider patients’ conditions mainly from the biomedical perspective. Meanwhile, nurses tend to consider their conditions mainly from psychosocial perspectives. The NP, as someone who can understand both contexts, acts as a bridge and works to improve the understanding of both doctors and nurses about patients. One participant stated:

“As a result of working with doctors, I realized that there is quite a gap between what nurses think and what doctors think, with seldom an intersection of thoughts.”

(NP12)

The quality of medical practice was improved by the NPs’ support for smooth communication between doctors and nurses. In addition, NPs provided nurses with medical knowledge for the proper understanding of nursing care. The NPs worked hard to create a safe and secure environment for nurses to work. One participant said:

“NPs must think about the quality of life in accordance with the disease or patient’s condition. If a person is paralyzed, there is a risk of falling, and if he/she is told to rest, there is a possibility of losing feeling in the body, so these persons should be actively moved. Of course, safety is important, but we should think of a plan to improve activities of daily living while ensuring safety and leave such decisions to the nurses.”

(NP6)

#### 3.3.2. Implementing Nursing Education Based on Flexible Contexts 

NPs acquire knowledge through practice and have more opportunities to use that knowledge while imparting education to other nurses. NPs’ experiences in the medical field, an opportunity that nurses rarely are given, have led to improved education for nurses.

“There are some things that the nurses in the operating room should know. They are unaware of things that I knew when I was a nurse, so I try to educate them or provide them input in the operating room.”

(NP12)

“When we work together, we talk to each other, report to the physicians, and make morning reports, and other nurses listen to what we say. I think these interactions can improve NPs’ and nurses’ understanding about physicians’ process of thinking.”

(NP2)

NPs working in the clinical setting not only improved the knowledge and skills of nurses but also enhanced their flexibility in terms of interprofessional collaboration. Japanese NPs can practice medicine after receiving training on medical treatment and are also responsible for educating NPs in the field. One participant stated:

“In our hospital, the training for specific activities started last year, and I oversee teaching and management of activities. I think that is where we can do more practical work than the administrators because they do not know much about NPs or other specific activities.”

(NP6)

### 3.4. Contribution to Healthcare 

#### 3.4.1. Team Perspective 

While working as NPs, participants were required to improve the quality of medical care from various perspectives. In addition to focusing on medical treatment, NPs looked for ways to contribute in their current capacity and thought about solutions to problems from a broad perspective.

“I think the most important thing is how you look at a problem, and then you need to be able to find the best way to solve it. It is important to identify a problem.”

(NP12)

“I think the most important point is to simply act as an assistant in a place where there is no one else, but … we are told that the more complicated the operation, the more we have to contribute. It is challenging to quantify these activities, but I think it is important.”

(NP3)

While collaborating with various professionals, it is important for NPs to be aware of the perspective of the entire team and to act to improve the quality of care from a safety perspective in relation to seemingly invisible problems.

#### 3.4.2. Exploring Ways to Improve the Quality of Healthcare 

NPs integrated medical and nursing contexts to practice direct patient care. In addition, they pursued benefits for patients and practiced innovative approaches. They identified problems in the healthcare system, found innovative solutions, and worked with other professionals outside the hospital to implement them.

“Not only do I work in the hospital, but there is also an affiliated facility, a special nursing home for the elderly, where I go with the doctor who provides home care once every 2 weeks. In the nursing home, healthcare professionals lack. So, when some of them have to support our procedures to patients there, they cannot provide assisted baths to the residents or help with the meals properly. In addition, bedridden residents no longer have to use wheelchairs for long periods, so I think that NPs’ participation in procedures in nursing homes is beneficial in healthcare.”

(NP7)

“As medical care becomes more advanced, the number of treatments available increases but what happens afterward has not been considered until now. I am now able to intervene in such areas.”

(NP4)

NPs have always been concerned about the best ways of providing medical care to patients. They try to contribute to the improvement of the healthcare system with respect to individual patients, the medical team, related facilities, and the community surrounding the patients.

### 3.5. Personal Qualities for Effective Working

#### 3.5.1. Motivation for Direct Patient Care

NPs emphasized the importance of focusing on direct patient involvement in a diverse work environment. NPs were involved in the direct care of the patients during their hospital stay, while maintaining their original nursing perspective and involvement in medical treatment from the broad perspective of an NP.

“I think we can provide medical care by utilizing the strengths of NPs, not only in terms of information from nurses but also from the perspective of nurses who are directly involved in the patient’s care.”

(NP11)

“What patients in Japan are looking for nowadays is probably someone in between a physician and a nurse, so that they can feel safe to see a doctor but also have a nurse who understands them and supports them in the absence of a doctor. I think patients would be most receptive to such a middle-management person.”

(NP2)

The NPs were directly involved in patient care, consciously working on the medical and nursing aspects of patient care, bridging patient care gaps from an intermediate position between physicians and nurses, and implementing care. They were more patient-friendly and required more time to provide care. The following statement shows that NPs were aware that this kind of treatment led to the patient’s sense of security.

“I think it is characteristic that we spend 30 min to 1 h per patient. I do not know, but I think people usually leave the room, saying thank you for the careful explanation. I think they say, ‘I was able to listen well,’ ‘I feel at ease,’ or ‘I’ll ask you again.’.”

(NP8)

#### 3.5.2. Contribution to Improving Quality of Life

The most important outcome that NPs sought in their work was improved quality of life for patients. To achieve this, NPs focused on direct interaction with patients, as they did when they were nurses, and incorporated theoretical thinking by utilizing their extensive medical knowledge. By doing so, they were able to build relationships with patients and gain the trust of multiple professionals.

“I think we have our own areas of interest, and the areas we are not interested in are the ones we need to work on. What we are interested in is the patient’s quality of life.”

(NP6)

Furthermore, to enhance the quality of life of patients, the participants were aware of not only their role in the acute care hospital but also of that in the community, and devised solutions so that the patients could lead their lives successfully in the community. One participant stated:

“As a community healthcare provider, I play the role of a coordinator for patients returning from the hospital to the community, from the acute care hospital to the convalescent care hospital.”

(NP4)

#### 3.5.3. Awareness of Being a Nurse 

While working as an NP, participants often had to perform tasks different from those of a nurse. However, NPs always remembered that they were nurses first and worked in a manner to leverage this perspective. They were aware of the similarities between the professions of NPs and nurses.

“It is essential to schedule everything because we do not get complete instructions from the physicians. We get to decide the treatment procedure for the patients based on our opinions as NPs and suggestions from the nurses about what is going on in the clinical setting.”

(NP4)

“Nursing lays the foundation for NPs. I think NPs are good in medical settings because they have the experience of seeing many patients every day. It is easy to detect abnormalities from small changes, such as a slight change in speech or behavior, or a lack of a smile.”

(NP11)

“When treating a patient, there is no difference between NPs and physicians in following the guidelines and prescribing medications or ordering laboratory tests. I am trying to bring out the best in the nurses.”

(NP8)

NPs’ awareness that they are fundamentally nurses prevents them from an identity crisis and allows them to focus on their duties, leading to an improvement in the quality of their work.

#### 3.5.4. Flexibility in Adapting to New Environments

For NPs to work effectively in the medical field, they needed to adapt their work style to different settings. The environment in which medical nurses work is diverse, and they are required to work flexibly by analyzing what is needed in each field using the competencies of medical nurses.

“I think the key words that describe NPs in general internal medicine are balance and flexibility. It is not necessary to specialize in anything, but I think that NPs who can respond flexibly to a wide range of problems are more likely to be of use.”

(NP1)

“Nowadays, I think that the roles of nurses and doctors are fixed to some extent, and they probably do not perform tasks outside their prescribed roles or have any new ideas because their work is already fixed. I think this opens an opportunity for medical nurses. I feel like I am filling this void.”

(NP6)

To work smoothly as an NP, it is necessary to constantly observe the environment in which one is placed, work flexibly based on competencies, and have a job description that allows for work in areas where other professionals might lack expertise.

## 4. Discussion 

This study clarified the present working conditions and perceptions of effective working styles of NPs in Japan and the essence of their work. Japanese expert NPs had established trusting relationships with physicians by understanding their context and provided comprehensive support for medical treatment based on this understanding. NPs’ medical knowledge and skills improved with experience, contributing to the education of doctors in training. Education was not limited to medical practice but was also focused on how to communicate with multiple professionals, which helped doctors in training assimilate into the medical field. As NPs added to their medical knowledge, they gained a deeper understanding of the risks involved in medical treatment and could identify the limits of their capabilities. By proactively communicating this risk to the doctors, the NPs displayed their awareness of medical safety and established clear roles that strengthened the trust of the doctors. In addition, NPs actively communicated with multidisciplinary teams with a modest attitude. By understanding the diverse contexts of each profession, they found gaps in communication and the means of addressing those gaps, thus acting as a bridge between multiple professions. In multidisciplinary teams, NPs shared treatment goals and practiced nursing without deviating from medical treatment plans. Simultaneously, they enhanced nursing education in clinical situations, aiming to raise the level of nursing competence and improve the quality of nursing care. NPs’ perspective for improving the quality of life of patients extended to the holistic environment of the patients. The NPs improved the healthcare system inside and outside the medical organizations. Furthermore, they were flexible and proactive in dealing with the problems that arose. 

Expert NPs in Japan have the potential to improve the quality of medical care by facilitating the work of physicians. Internationally, several studies have evaluated the activities of NPs in terms of collaboration with physicians [19]. This study showed that efficient collaboration with physicians can foster effective patient care. Miranda and colleagues [9] conducted a systematic review of studies on the quality of primary care practice of NPs as an alternative to physicians and concluded that the quality of NP practice is equal to or better than that of physicians. Norful reported that physicians and nurses saw nurse–physician substitution and collaboration as a way of augmenting people’s access to care and improving the quality and continuity of care [20]. Contrastingly, as this study shows, NPs may not be understood well in Japanese clinical settings. Efforts to promote effective NP–physician management should be supported in clinical practice. Thus, effective collaboration between NPs and physicians should comprise an understanding of physicians’ contexts by NPs as well as physicians’ support of NPs. Furthermore, owing to a lack of doctors in Japan, the increased workload has resulted in delays in patient treatment. Having NPs perform medical activities in the same context as physicians contributes to the timely provision of quality treatment to patients and reinforces the appropriateness of treatment.

NPs working with physicians and understanding their context also fosters interprofessional collaboration. Information sharing within the team is essential for promoting effective medical care. However, multidisciplinary teams are often unable to share treatment plans promptly. As this study shows, interventions to facilitate NPs’ communication can be critical to improve their interprofessional collaboration. The Institute of Medicine stated that medicine is not necessarily a team sport. In a successful high-functioning team model, the following personal values are important: (1) honesty and transparency, (2) self-control, (3) creativity, (4) humility, and (5) curiosity [21]. Even when NPs become experts, they do not lead teams in medicine but contribute to the promotion based on mutual respect for medical professions. This study shows that NPs can become effective transmitters of physicians’ ideas about patients to other medical professionals. Ito stated that NPs in Japan realize that they are the bridge between multiple professions and coordinate with the team respectfully by interacting with multiple professionals [16]. There has been an emphasis on interprofessional collaboration without hierarchy among medical care professionals [22]. In Japan, there is a professional hierarchy between physicians, nurses, care workers, and care managers [23,24,25]. In this hierarchy, physicians’ opinions are more strongly recognized than those of other professionals regarding decision-making on patient care. Through communication, NPs can mitigate this hierarchical relationship by bridging the understanding about patients among the professionals.

Japanese expert NPs were aware that humility and cooperation were important in communicating with multiple professionals and to be accepted as medical professionals. The current participants had 4–10 years of experience as NPs, and the term “humility”, as well as respect for others, was essential to their work. NPs tend to embrace humility, which suggests an acute awareness of the need to control arrogance. The Japanese healthcare system tends to emphasize organizational activities because of its historical collectivist background [26]. It is believed that for an organization to be cohesive, NPs must suppress self-assertion and maintain harmony in medical teams.

This study showed that Japanese NP experts considered their standpoints in their teams and organizations and adjusted their opinions and collaboration with other professionals, including physicians. Building good relationships with doctors and staff is conducive to teamwork worldwide [27]. Given Japan’s background of fostering a culture of organizational activities, Japanese NPs’ humble behavior within the team may further provide an exemplary model of interprofessional collaboration. The collaboration of NPs with nurses, who are the primary constituents of medical teams, is critical.

In the early days of NPs in the US, the role of the NP was developed to attract nurses away from nursing and into medicine, thereby undermining the unique role of nursing in healthcare [28]. With the accumulation of evidence and the conceptualization of NPs, they are now positioned as advanced practice nurses [29], suggesting that NPs do not deviate from nursing practice and instead provide a more developed model of nursing. In this study, findings suggested that Japanese NP experts tried to respect the standpoints of nurses in clinical settings and act based on nurses’ perspectives. While professional nurses play a significant role in improving the quality of nursing by pursuing its unique roles and activities, NPs improve the quality of nursing by working with the team to provide direct holistic nursing care to patients. NPs also contribute to improving the competence of clinical nurses [30]. As this study shows, Japanese experts collaborated with hospital nurses on common grounds and transferred medical information to nurses. Currently, nurses in clinical practice in Japan are faced with the anxiety of having to care for several patients with diverse diseases and different clinical situations simultaneously, while consistently updating their medical knowledge [31]. NPs can effectively mitigate nurses’ workload and understand that the anxiety may be influenced by the nurses’ cognitive load about medicine [32]. Nurses operate in a different work environment than physicians and have limited opportunities to gain a deeper understanding of various treatments and laboratory testing. When NPs appropriately communicate medical contexts to nurses and provide consultation on nursing care, nurses acquire medical knowledge and feel secure, ultimately contributing to improving the quality of nursing care. As the physicians’ context needs to be well understood, NPs must be free from general nursing duties while also being given autonomy in collaborating with physicians in disease management. It is only from a relative position of independence that NPs can intervene to provide macro and micro perspectives and evolve nursing care. NPs contribute to the management of nursing care at the frontline level, which differs from the role of top nurse managers, who adopt a holistic perspective [33].

NPs contribute to various aspects of healthcare. As science and technology are changing rapidly, social values are also changing and becoming highly diverse. In improving the quality of healthcare, stakeholders must be responsive to this change and diversity while maintaining the universal essence of medicine. In this study, NPs suggested that they can facilitate this improvement in healthcare quality through the integrated advancement of medicine in community care and health promotion. The World Health Organization states that quality of care refers to the extent to which health services for individuals and populations increase the likelihood of desired health outcomes [34]. In particular, quality care is based on evidence-based expertise; is effective; avoids harm to the population under care; responds to individual preferences, needs, and values; and is achieved through timely and equitable healthcare delivery systems; furthermore, holistic healthcare heals and supports, and utilizes efficient organizational systems that maximize the benefits accrued from resources [35]. There are many problems in clinical practice, such as doctor shopping, owing to distrust of medical care, and care for the elderly, where advanced medical care is prioritized over quality of life [36]. As this study shows, expert NPs identify the problems from the perspective of the entire team and take measures to overcome them. Apart from filling in the gaps within the team, these NPs ensure the quality of healthcare for patients, incorporate their creative ideas into reality, and work to solve problems as an implementation unit. Japanese expert NPs need to be encouraged by their teams and organizations to be active inside and outside the organization. Globally, there are restrictions on the activities of NPs owing to organizational and governmental and local policies [37]. As NPs in Japan are not nationally legally certified, the responsibility for their activities falls on the organizations that employ them [38,39]. In rapidly aging societies, there is a requirement for a new entity that is flexible. Japanese NPs can fill this void in clinical settings by being educated in the Japanese context [40,41,42]. Training NPs to reflect on the Japanese context is the basis of education, and an increase in the number of such NPs will lead to improved healthcare in Japan. Nursing education has improved through the application of clinical ladder as a competency-based education, based on various nursing contexts [43,44]. Furthermore, it is well known that multidisciplinary team collaboration is beneficial worldwide [45]. As this research and another study showed, the Japanese NP approach is effective for multidisciplinary collaboration and may be applicable in countries where healthcare requirements are complex [46]. Regarding international contexts, Japanese NPs should develop competencies that fit NP roles in different clinical settings.

### Limitations 

A limitation of this study was credibility. As the first author is an NP and has been involved in NP training, there was a possibility of cognitive bias in recruiting NPs. To improve the quality of the credibility, we received guidance from a physician who was familiar with qualitative research. The quality of this study was also maintained because the data were saturated by Japanese nurse practitioners and general physicians to ensure dependability. Another limitation is transferability. This study included few primary care NPs because few Japanese NPs specialize in primary care. This study was also conducted with expert NPs in the Japanese context and may not be adaptable to other cultures, limiting transferability. This study focused on the comprehensive competencies of Japanese expert NPs. Transferability of this study was maintained because we explored comprehensive competencies across domains and describe the background of Japanese NPs. Future studies should be conducted in other settings and by domain, primary and critical, respectively. Furthermore, future studies should focus on the competencies necessary for NPs in Japanese and other Asian contexts to improve the quality of education for NPs.

## 5. Conclusions 

This study showed that Japanese expert NPs function effectively in medical settings by collaborating flexibly and humbly with various professionals who also have autonomous positions. They can also contribute to the improvement of the quality of healthcare by proposing practical solutions to problems faced by patients and medical organizations, and by implementing the solutions using their capabilities as nurses and through creative ideas. These effective competencies of Japanese expert NPs can be applied to other similar sociocultural contexts, such as in other Asian countries, and can be used to define new competencies and legally secured job descriptions in the context of Japanese NPs.

## Figures and Tables

**Table 1:** Identified competencies and roles among Japanese expert nurse practitioners.

Theme	Concepts
Working in physicians’ contexts	Understanding the physician context
	Comprehensive support for medical practice
	Active involvement in physician education
	Knowing the limits of one’s capabilities
Interprofessional collaboration	Bridging multiple professions
	Taking advantage of diverse contexts
	Humble collaboration
Involvement in nurses’ working	Communicating medical contexts
	Implementing nursing education based on flexible contexts
Contribution to healthcare	Team perspective
	Exploring ways to improve the quality of healthcare
Personal qualities for effective working	Motivation for direct patient care
	Contribution to improving quality of life
	Awareness of being a nurse
	Flexibility in adapting to new environments

## Data Availability

All relevant datasets in this study are described in the manuscript.
